# Validated imaging biomarkers as decision-making tools in clinical trials and routine practice: current status and recommendations from the EIBALL* subcommittee of the European Society of Radiology (ESR)

**DOI:** 10.1186/s13244-019-0764-0

**Published:** 2019-08-29

**Authors:** Nandita M. deSouza, Eric Achten, Angel Alberich-Bayarri, Fabian Bamberg, Ronald Boellaard, Olivier Clément, Laure Fournier, Ferdia Gallagher, Xavier Golay, Claus Peter Heussel, Edward F. Jackson, Rashindra Manniesing, Marius E. Mayerhofer, Emanuele Neri, James O’Connor, Kader Karli Oguz, Anders Persson, Marion Smits, Edwin J. R. van Beek, Christoph J. Zech, European Society of Radiology

**Affiliations:** 1 Cancer Research UK Imaging Centre, The Institute of Cancer Research and The Royal Marsden Hospital, Downs Road, Sutton, Surrey, SM2 5PT UK; 2 Ghent University Hospital, Ghent, Belgium; 3 QUIBIM SL / La Fe Health Research Institute, Valencia, Spain; 4 Department of Radiology, University of Freiburg, Freiburg im Breisgau, Germany; 5 VU University Medical Center, Amsterdam, The Netherlands; 6 Hopital Européen Georges Pompidou, Paris, France; 7 University of Cambridge, Cambridge, UK; 8 UCL Institute of Neurology, London, UK; 9 Universitätsklinik Heidelberg, Translational Lung Research Center (TLRC), German Center for Lung Research (DZL), University of Heidelberg, Im Neuenheimer Feld 156, 69120 Heidelberg, Germany; 10 University of Wisconsin School of Medicine and Public Health, Madison, WI USA; 11 Department of Radiology and Nuclear Medicine, Radboud University Medical Center, Geert Grooteplein 10, 6525 GA Nijmegen, The Netherlands; 12 Medical University Vienna, Vienna, Austria; 13 Department of Translational Research, University of Pisa, Pisa, Italy; 14 Division of Cancer Sciences, University of Manchester, Manchester, UK; 15 Hacettepe University Hospitals, Ankara, Turkey; 16 Linköpings Universitet, Linköping, Sweden; 17 Department of Radiology and Nuclear Medicine (Ne-515), Erasmus MC, PO Box 2040, 3000 CA Rotterdam, The Netherlands; 18 Edinburgh Imaging, Queen’s Medical Research Institute, Edinburgh Bioquarter, 47 Little France Crescent, Edinburgh, UK; 19 University Hospital Basel, Radiology and Nuclear Medicine, University of Basel, Petersgraben 4, CH-4031 Basel, Switzerland; 20 European Society of Radiology, Am Gestade 1, 1010 Vienna, Austria

**Keywords:** Imaging biomarkers, Clinical decision making, Quantitation, Standardisation

## Abstract

Observer-driven pattern recognition is the standard for interpretation of medical images. To achieve global parity in interpretation, semi-quantitative scoring systems have been developed based on observer assessments; these are widely used in scoring coronary artery disease, the arthritides and neurological conditions and for indicating the likelihood of malignancy. However, in an era of machine learning and artificial intelligence, it is increasingly desirable that we extract quantitative biomarkers from medical images that inform on disease detection, characterisation, monitoring and assessment of response to treatment. Quantitation has the potential to provide objective decision-support tools in the management pathway of patients. Despite this, the quantitative potential of imaging remains under-exploited because of variability of the measurement, lack of harmonised systems for data acquisition and analysis, and crucially, a paucity of evidence on how such quantitation potentially affects clinical decision-making and patient outcome. This article reviews the current evidence for the use of semi-quantitative and quantitative biomarkers in clinical settings at various stages of the disease pathway including diagnosis, staging and prognosis, as well as predicting and detecting treatment response. It critically appraises current practice and sets out recommendations for using imaging objectively to drive patient management decisions.

## Key points

- Biomarkers derived from medical images inform on disease detection, characterisation and treatment response.
- Quantitative imaging biomarkers have potential to provide objective decision-support tools in the management pathway of patients.
- Measurement variability needs to be understood and systems for data acquisition and analysis harmonised before using quantitative imaging measurements to drive clinical decisions.

## Introduction

**Fig. 1:**
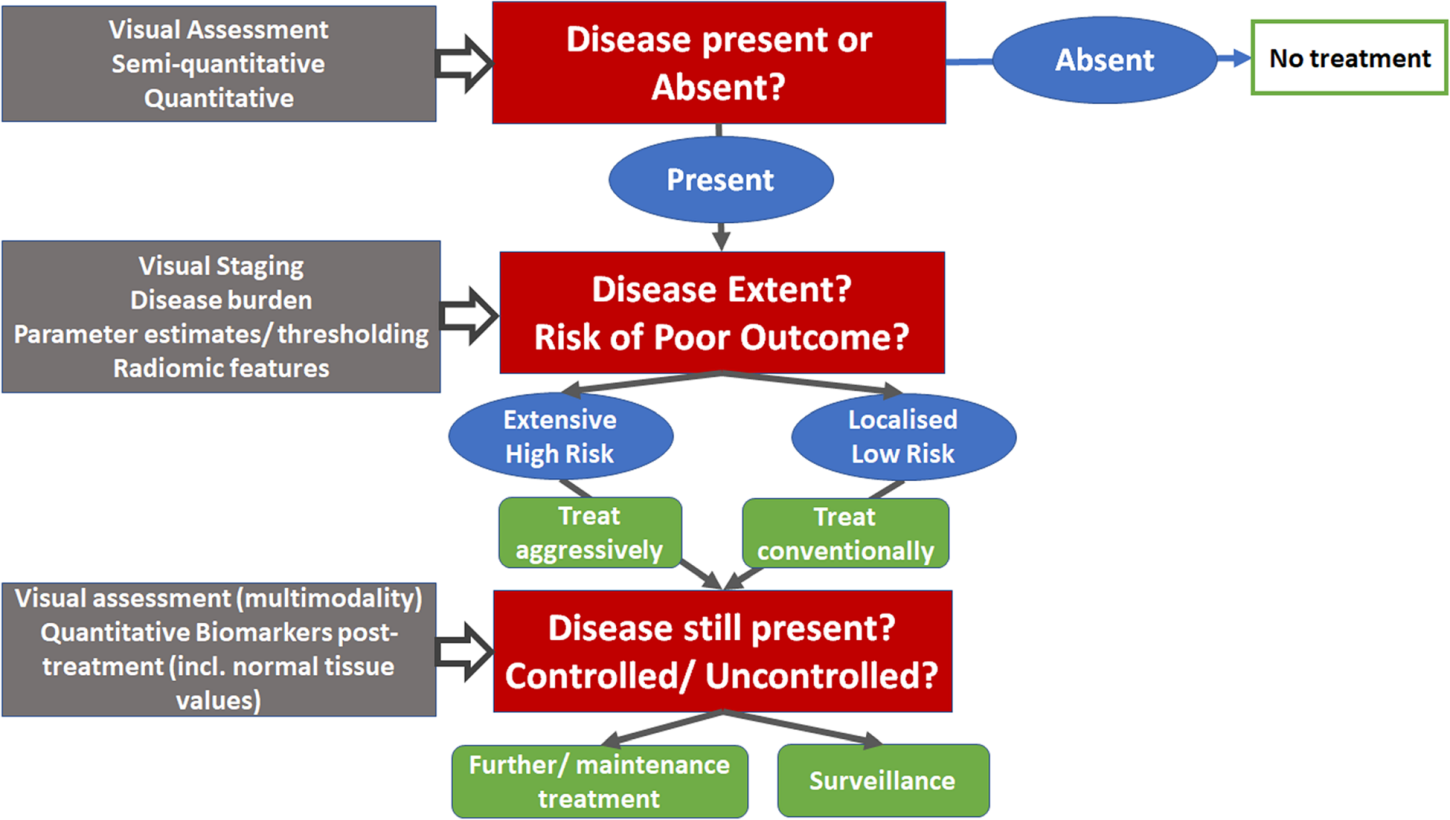
Schematic of questions requiring decisions (red boxes), imaging assessments (grey boxes), the results of the imaging assessments (blue ovals) and the management decisions they potentially influence (green boxes)

Interpretation of medical images relies on visual assessment. Accumulated and learnt knowledge of anatomical and physiological variations determines recognition of appearances that are within “normal limits” and allows a pathological change in appearances outside these limits to be identified. Observer-driven pattern recognition dominates the way that imaging data are used in routine clinical practice (Fig. 1). A semi-quantitative approach to image analysis has been advocated in various scenarios. These use observer-based categorical scoring systems to classify images according to the presence or absence of certain features. Examples used widely in healthcare for clinical decision-making include reporting and data systems (RADS) [1, 2]. Increasingly, however, advancement in standardisation efforts, applications of analysis techniques to extract quantitative information and machine and deep learning techniques are transforming how medical images may be exploited.

In some clinical scenarios, automated quantitation may be more objective and accurate than manual assessment; thresholds can be applied above or below which a disease state is recognised and subsequent changes interpreted as clinically relevant [3]. Unlike biomaterials, images potentially can be transferred worldwide easily, cheaply and quickly for biomarker extraction in an automated, reproducible and blinded manner. Nevertheless, despite the substantial advantages of quantitation, very few quantitative imaging biomarkers are used in clinical decision-making due to several obstacles. Harmonisation of data acquisition and analysis is non-trivial. Lack of international standards without routine quality assurance (QA) and quality control (QC) processes results in poorly validated quantitative biomarkers that are subject to errors in interpretation [4–6]. This has profound implications for diagnosis (correct interpretation of the presence of the disease state) [7] and treatment decision-making (based on interpretation of response vs non-response) [8] and reduces the validity of combination biomarkers derived from hybrid (multi-modality) imaging systems. The imaging community needs to engage in delivering high-quality data for quantification and adoption of machine learning to ultimately exploit quantitative imaging information for clinical decision-making [9]. This manuscript describes the current evidence and future recommendations for using semi-quantitative or quantitative imaging biomarkers as decision-support tools in clinical trials and ultimately in routine clinical practice.

## Validated imaging biomarkers currently used to support clinical decision-making

The need for absolute quantitation (versus semi-quantitative assessment) in decision-making should be clearly established. Absolute quantitation is demanding and resource intensive because hardware and software differences across centres and instrumentation and their evolution impact the quality of quantified data. Rigorous on-going QA and QC are essential to support the validity and clinically acceptable repeatability of the measurement, and efforts are on-going within RSNA and the ESR and other academic societies. Critically also, definitive thresholds to confidently separate normal from pathological tissues based on absolute quantitative metrics often do not have wide applicability or acceptance.

### Semi-quantitative scoring systems

Semi-quantitative readouts of scores based on an observer-recognition process are widely used because visual interpretation often has proven adequate and is linked to outcome. For example, MRI scoring systems for grading hypoxic-ischaemic injury in neonates using a combination of T1-weighted (T1W) imaging, T2-weighted (T2W) imaging and diffusion-weighted imaging (DWI) have shown that higher post-natal grades were associated with poorer neuro-developmental outcome [10]. In cervical spondylosis, grading of high T2-weighted (T2W) signal within the spinal cord has been related variably to disease severity and outcome [11, 12]. In common diseases such as osteoarthritis, where follow-up scans to assess progression are vital in treatment decision-making, such scoring approaches also are useful [13]; web-based knowledge transfer tools using the developed scoring systems indicate good agreement between readers with both radiological and clinical background specialisms in interpreting the T2W MRI data [14]. Similar analyses have been extensively applied in diseases such as multiple sclerosis [15] and even to delineate the rectal wall from adjacent fibrosis [16]. In cancer imaging, ^18^FDG PET/CT studies use the Deauville scale (liver and mediastinum uptake as reference) as the standard for response assessment in lymphoma [17]. Semi-quantitative scoring systems also form the basis of the breast imaging (BI)-RADS and prostate imaging (PI)-RADS systems in breast and prostate cancer respectively. Their wide adoption has led to spawning of similar classification scores for liver imaging (LI)-RADS [18–20], thyroid imaging (TI)-RADS [20] and bladder (vesicle imaging, VI)-RADS [21] tumours. Multiparametric MRI scores are also used for detection of recurrent gynaecological malignancy [22] and grading of renal cancer [23]. Manual assessment of lung nodule diameter and volume doubling time have reached a wide acceptance in the decision-making of incidental detection, screening [24] and prediction of response [25]. These parameters might be substituted or improved by artificial intelligence in the near future [26].

### Quantitative measures of size/volume

The simplest quantitative measure used routinely is size. Size is linked to outcome in both non-malignant and malignant disease [27]. Ventricular size on echocardiography is robust and incorporated into large multicentre trials [28, 29] and into routine clinical care. Left ventricular ejection fraction (LVEF) is routinely extracted from both ultrasound and MRI measurements. In inflammatory diseases such as rheumatoid arthritis, where bone erosions are a central feature, assessment of the volume of disease on high-resolution CT provides a surrogate marker of disease severity [30] and is associated with the degree of physical impairment and mortality [31, 32]. Yet these methods remain to be implemented in a clinical setting because intensive segmentation and post-processing resources are required. In cancer studies, unidimensional measurements (RECIST1.0 and 1.1) [27] are used for response because of the perceived robustness and simplicity of the measurement, although reproducibility is variable [33], resulting in uncertainty [34]. Although numerous studies have linked disease volume to outcome over decades of research [35–38], volume is not routinely documented in clinical reports because of the need for segmentation of irregularly shaped tumours. Volume is indicative of prognosis and response, for example in cervix cancer where evidence is strong [39]. In other cancer types, such as lung, metabolic active tumour volume on PET has a profound link to survival [40, 41]. Metabolic active tumour volume also has proven to be a prognostic factor in several lymphoma studies [42] and is being explored as a biomarker for response to treatment [43–45]. The availability of automated volume segmentation at the point of reporting is essential for routine adoption.

## Extractable quantitative imaging biomarkers with potential to support clinical decision-making

Quantitative imaging biomarkers that characterise tissue features (e.g. calcium, fat and iron deposition, cellularity, perfusion, hypoxia, diffusion, necrosis, metabolism, lung airspace density, fibrosis) can provide information that characterises a disease state and reflects histopathology. Multiple quantitative features can be incorporated into algorithms for recognising disease and its change over time (both natural course and in response to therapy). This involves an informatics style approach with data built from atlases derived from validated cases. Curation of anatomical databases annotated according to disease presence, phenotype and grade can then be used with the clinical data to build predictive models that act as decision-support tools. This has been proposed for brain data [46] but requires a collection of good quality validated data sets, carefully archived and curated. Harnessing the quantitative information contained in images with rigorous processes for acquisition and analysis, together with deep-learning algorithms such as has been demonstrated for brain ageing [47] and treatment response [48], will provide a valuable decision-support framework.

### Ultrasound

Quantitation in ultrasound imaging has derived parameters related to cardiac output (left ventricular ejection fraction), tissue stiffness (elastography) and vascular perfusion (contrast-enhanced ultrasound) where parameters are related to blood flow. Ultrasound elastography is an emerging field; it has been shown to differentiate liver fibrosis [49], benign and malignant breast and prostate masses and invasive and intraductal breast cancers [50, 51]. It also has been explored for quantifying muscle stiffness in Parkinson’s disease [52], where low interobserver variation and significant differences in Young’s modulus between mildly symptomatic and healthy control limbs make it a useful assessment tool. Furthermore, it has shown acceptable inter-frame coefficient of variation for identifying unstable coronary plaques [53]. Blood flow quantified by power Doppler has potential as a bedside test for intramuscular blood flow in the muscular dystrophies [54]. Quantified parameters peak intensity (PI), mean transit time (MTT) and time to peak (TTP) are available from contrast-enhanced ultrasound, but rarely used because of competing studies with CT and MRI that also capture morphology.

### CT

CT biomarkers are dependent on a single biophysical parameter, differential absorption of X-rays due to differences in tissue density, either on unenhanced scans or following administration of iodine-based contrast agent, which increases X-ray absorption in highly perfused tissues. Other developments have utilised tissue density as a parameter in multicentre trials for quantification of emphysema (COPDGene and SPIROMICS) [55–57] and interstitial pulmonary fibrosis (IPF-NET) [58] and for assessment of obstructive (reversible) airways disease [59, 60]. The studies have made use of various open source and bespoke research software tools, but generally, these imaging-based biomarkers have been used to guide treatment [61, 62] and demonstrated direct correlation with outcomes and functional parameters [63]. Drawbacks include poor standardisation of imaging protocols (voltage, slice thickness, respiration, I.V. contrast, kernel size) and post-processing software [64], although many of these issues have been resolved using phantom quality assurance and specified imaging procedures for every CT system used in these studies [65, 66]. Standardisation of instrumentation would simplify comparability between centres and enable long-term data acquisition consistency even after scanner updates [66]. In cardiac imaging, tissue density biomarkers using coronary artery calcium scoring have been extensively applied in large studies evaluating cardiac risk [67] and luminal size on coronary angiography used in outcome studies [68, 69]. Dual-energy CT quantifies iodine concentration directly and is being investigated for characterising pulmonary nodules and pleural tumours [70, 71].

### MR including multiparametric data

MRI is more versatile than US and CT because it can be manipulated to derive a number of parameters based on multiple intrinsic properties of tissue (including T1- and T2 relaxation times, proton density, diffusion, water-fat fraction) and how these are altered in the presence of other macromolecules (e.g. proteins giving rising to magnetisation transfer and chemical exchange transfer effects) and externally administered contrast agents (Gadolinium chelates). Perfusion metrics have also been derived with arterial spin labelling, which does not require externally administered agents [72]. The apparent diffusion coefficient (ADC) is the most widely used metric in oncology for disease detection [73, 74], prognosis [75] and response evaluation [76, 77]. Post-processing methods to derive absolute quantitation are extensively debated [78, 79], but the technique is robust with good reproducibility in multicentre, multivendor trials across tumour types [80]. Refinements to model intravascular incoherent motion (IVIM) and diffusion kurtosis are currently research tools. In cardiovascular MRI, there is a growing interest in quantifying T1 relaxation time, rather than just relying on its effect on image contrast; when combined with the use of contrast agents, T1 mapping allows investigation of interstitial remodeling in ischaemic and non-ischaemic heart disease [81]. T1 values are useful to distinguish inflammatory processes in the heart [82], multiple sclerosis in the central nervous system [83], iron and fat content in the liver [84, 85] and adrenal [86], which correlates with fibrosis scores on histology [87]. Multiparametric MRI biomarkers (T1 and proton density fat fraction) achieve a > 90% AUC for differentiating patients with significant liver fibrosis and steatosis on histology [88] and are being supplemented by measurements of tissue stiffness (MR elastography) where a measurement repeatability coefficient of 22% has been demonstrated in a metaanalysis [89]. Chemical exchange saturation transfer (CEST) MRI interrogates endogenous biomolecules with amide, amine and hydroxyl groups; exogenous CEST agents such as glucose provide quantitative imaging biomarkers of metabolism and perfusion. Quantitative CEST imaging shows promise in assessing cerebral ischaemia [90], lymphedema [91], osteoarthritis [92] and metabolism/pH of solid tumours [93]. However, the small signal requires higher field strength acquisition and substantial post-processing.

### Positron emission tomography (PET)-SUV metrics

Quantitation of ^18^FDG PET/CT studies is mainly performed by standardised uptake values (SUVs), although other metrics such as metabolic active tumour volume (MATV) and total lesion glycolysis are being introduced in studies and the clinic [94, 95]. The most frequently used metric to assess the intensity of FDG accumulation in cancer lesions is, however, still the maximum SUV. SUV represents the tumour tracer uptake normalised for injected activity per kilogram body weight. SUV and any of the other PET quantitative metrics are affected by technical (calibration of systems, synchronisation of clocks and accurate assessment of injected ^18^FDG activity), physical (procedure, methods and settings used for image acquisition, image reconstruction and quantitative image analysis) and physiological factors (FDG kinetics and patient biology/physiology) [96]. To mitigate these factors, guidelines have been developed in order to standardise imaging procedures [96, 97] and to harmonise PET/CT system performance at a European level [97, 98]. Newer targeted PET agents are only assessed qualitatively on their distribution (Table 1).

**Table 1:** Imaging biomarkers for disease detection (semi-quantitative and quantitative) with examples of current evidence for their use that would support decision-making

Disease detection							
	Biomarker	SemiQ/Q	Disease	Question answered	Utility of biomarker	Data from	Potential decision for
Non-malignant disease	**LVEF-US** **LVEF-MRI**	Q	Cardiac function [28, 29] Cardiac function	Cardiac output Cardiac output	ICC US 0.72, single centre sensitivity 69% [29] ICC MRI 0.86,correlation of MRI and cineventriculography 0.72 [99]	Single centre US Multicentre MRI [99, 100]	Inotropes Inotropes
	Renal volume-US, CT, MRI	Q	Renal failure	Mass of parenchyma	ICC on US 0.64–0.86 [101] Correlation of US with CT 0.76–0.8 [102] Interobserver reproducibility on MRI 87–88% [103]	Single centre	Renal replacement, safety and toxicity of other pharmaceuticals
	Young’s modulus on elastography-US	Q	Thyroid [104], breast [50] and prostate cancer [51] Parkinson’s disease	Tumour presence Muscle stiffness	Thyroid sensitivity 80%, specificity 95% [104] Breast AUC 0.898 for conventional US, 0.932 for shear wave elastography, and 0.982 for combined data [105] Prostate sensitivity 0.84, spec 0.84 [51]	Thyroid, breast: single centre Prostate meta-analysis	Treatment with surgery/radiotherapy/chemotherapy
	Lung tissue density	Q	Emphysema [106, 107] and fibrosis [58]	Airways obstruction, interstitial lung disease present	Emphysema (density assessment) influences BODE (body mass index, airflow obstruction, dyspnea and exercise capacity) index. Odds ratio of interstitial lung abnormalities for reduced lung capacity 2.3	Multicentre Single centre	Surgery, valve and drug treatment
	Fibrosis and ground-glass index on CT lung	SQ	Idiopathic lung fibrosis	Development of inflammation and fibrosis	Mortality predicted by pulmonary vascular volume (HR 1.23 (1.08–1.40), *p* = 0.001) and honeycombing (HR 1.18 (1.06–1.32), *p* = 0.002) [108]	Single centre	Drug treatment
	ADC/pCT	SQ	Ischaemic stroke	Presence of salvageable tissue versus infarct core	Measure of infarct core/penumbra used for patient stratification for research [109]	Planned multicentre	Treatment
Malignant disease	Lung RADS, PanCan, NCCN criteria [110, 111]	SQ	Lung nodules	Risk of malignancy	AUC for malignancy 0.81–0.87 [110]	Multicentre	Time period of follow-up or surgery
	CT blood flow, perfusion, permeability metrics	Q	Malignant neck lymph nodes Hepatocellular cancer	Tumour presence	Sensitivity 0.73, specificity 0.70 [112] AUC 0.75, sensitivity 0.79, specificity 0.75 [113]	Single centre Single centre	Staging and management (surgery, radiotherapy or chemotherapy)
	*BI-RADS* [114] *PI-RADS* [115] LI-RADS [116]	SQ	Cancer	Risk of malignancy	PPV: BI-RADS0 14.1 %, BI-RADS4 39.1 % and BI-RADS5 92.9 % PI-RADS2 pooled sensitivity 0.85, pooled specificity 0.71 Pooled sensitivity for malignancy 0.93	Dutch breast cancer screening programme Meta-analysis Systematic review	Staging and management stratification (surgery, radiotherapy, chemotherapy, combination)
	*ADC*	Q	Cancer [117] Liver lesions [118] Prostate cancer [119]	Tumour presence	Liver AUC 0.82–0.95 Prostate AUC 0.84	Single centre Single centre	Staging and management stratification (surgery, radiotherapy, chemotherapy, combination)
	Dynamic contrast enhanced metrics (K^trans^, K_ep_, blood flow, V_e)_	Q	Liver tumour Recurrent glioblastoma		Hepatocellular cancer AUC 0.85, sensitivity 0.85, specificity 0.81 [113] Brain- K^trans^Accuracy 86% [120]	Single centre Single centre	Further treatment
	^*18*^ *FDG SUV*	Q	Cancer Sarcoma [121] Lung cancer [105]	Tumour presence	Sarcoma—sensitivity 0.91, specificity 0.85, accuracy 0.88 Lung—sensitivity 0.68 to 0.95 depending on histology	Meta-analysis Meta-analysis	Staging and management stratification (surgery, radiotherapy, chemotherapy, combination)
	Targeted radionuclides [122]In-octreotide [123] [68]Ga DOTATOC and [68]Ga DOTATATE [124, 125] [68]Ga PSMA [4]	Non-Q	Cancer	Tumour presence	Sensitivity 97% and specificity 92% for octreotide [126] Sensitivity 100% and specificity 100% for PSMA [127]	Single centre Single centre	Validation remains difficult because of biopsying multiple positive sites.

Biomarkers used visually in the clinic are given in italics, and those that are used quantitatively are in bold

*Abbreviations*: *ADC* apparent diffusion coefficient, *APT* amide proton transfer, *AUC* area under curve, *BI-RADS* breast imaging reporting and data systems, *CBV* cerebral blood volume, *CoV* coefficient of variation, *CR* complete response, *CT* computerised tomography, *DCE* dynamic contrast enhanced, *DFS* disease-free survival, *DOTATOC* DOTA octreotitide, *DOTATATE* DOTA octreotate, *DSC* dynamic susceptibility contrast, *ECG* electro cardiogram, *FDG* fluorodeoxyglucose, *FLT* fluoro thymidine, *HR* hazard ratio, *HU* Hounsfield unit, *ICC* intraclass correlation, *IQR* interquartile range, *LVEF* left ventricular ejection fraction, *MRF* magnetic resonance fingerprinting, *MRI* magnetic resonance imaging, *MTR* magnetisation transfer ratio, *NCCN* National Comprehensive Cancer Network, *OS* overall survival, *pCT* perfusion computerised tomography, *PERCIST* positron emission tomography response criteria in solid tumours, *PD* progressive disease, *PFS* progression-free survival, *PPV* positive predictive value, *PI-RADS* prostate imaging reporting and data systems, *PR* partial response, *PSMA* prostate-specific membrane antigen, *RECIL* response evaluation in lymphoma, *RECIST* response evaluation criteria in solid tumours, *ROC* receiver operating characteristic, *SD* stable disease, *SUV* standardised uptake value, *SWE* shear wave elastography, *US* ultrasound

### Radiomic signature biomarkers

Radiomics describes the extraction and analysis of quantitative features from radiological images. The assumption is that radiomic features reflect pathophysiological processes expressed by other “omics”, such as genomics, transcriptomics, metabolomics and proteomics [128]. Hundreds to thousands of radiomic features (mathematical descriptors of texture, heterogeneity or shape) can be extracted from a region or volume of interest (ROI/VOI), derived manually or semi-automatically by a human operator, or automatically by a computer algorithm. The radiomic “signature” (summary of all features) is expected to be specific for a given patient, patient group, tissue or disease [129, 130]: it depends on the type of imaging data (CT, MRI, PET) and is influenced by image acquisition parameters (e.g. resolution, reconstruction algorithm, repetition/echo times for MRI), hardware (e.g. scanner model, coils), VOI/ROI segmentation [131] and image artifacts.

Unlike biopsies, radiomic analyses, although not tissue specific, capture heterogeneity across the entire volume [132], potentially making them more indicative of therapy response, resistance and survival. They may be therefore better suited to decision support in terms of treatment selection and risk stratification. Current radiomics research in X-ray mammography [133] and cross-sectional imaging (lung, head and neck, prostate, GI tract, brain) has shown promising results [134], leading to extrapolation in non-malignant disease. Image quality optimisation and standardisation of data acquisition are mandatory for widespread application. At present, individual research groups derive differing versions of a similar signature and there is a tendency to change the signature from study to study. Since radiomic signatures are typically multi-dimensional data, they are an ideal input for advanced machine learning techniques, such as artificial neural networks, especially when big multicentric datasets are available. Early reports from multicentre trials indicate that reproducibility of feature selection is good when extracted from CT [135] as well as MRI [136] data.

## Selecting and translating appropriate imaging biomarkers to support clinical decision-making

Automated quantitative assessments rather than scoring systems are easier to incorporate into artificial intelligence systems. For this, threshold values need to be established and a probability function of the likelihood of disease vs. no disease derived from the absolute quantitation (e.g. bone density measurements) [137]. Alternatively, ratios of values to adjacent healthy tissue can be used to recognise disease. Similarly, for prognostic information, thresholds established from large databases will define action limits for altering management based on the likelihood of a good or poor outcome predicted by imaging data. This will enable the clinical community to move towards using imaging as a “virtual biopsy”. The current evidence for use of quantitative imaging biomarkers for diagnostic and prognostic purposes is given in Tables 1 and 2 respectively.

**Table 2:** Imaging biomarkers for disease characterisation (semi-quantitative and quantitative) with examples of current evidence for their use that would support decision-making

	Biomarker	SemiQ/Q	Disease	Question answered	Utility of biomarker	Data from	Potential decision for
Non-malignant disease	Young’s modulus	Q	Coronary plaques [53]	Risk of rupture	Reproducibility CoV 22% vessel wall, 19% in plaque. AUC for focal neurology Youngs modulus + degree = 0.78	Single centre	Stenting, coronary bypass surgery
	Plaque density, vessel luminal diameter	Q	Coronary artery stenosis	Risk of plaque rupture; risk of significant cardiac ischaemia, infarction, death	No luminal narrowing but with coronary artery calcium (CAC) score > 0 had a 5-year mortality HR 1.8 compared with those whose CACS = 0. No luminal narrowing but CAC ≥ 100 had mortality risks similar to individuals with non-obstructive coronary artery disease [138] CT angiography significantly better at predicting events than stress echo/ECG [68] Coronary death/non-fatal myocardial infarction was lower in patients with stable angina receiving CT angiography than in the standard-care group (HR = 0.59) [69]	Multicentre Multicentre Multicentre	Statins, stenting, coronary bypass surgery
	^18^F-Na	SQ	Aortic valve disease Coronary plaque [139] Acute events from abdominal aortic aneurysm	Valve stenosis present Likelihood of plaque rupture Likelihood of aneurysm rupture	Reproducibility NaF uptake 10% [140] Baseline 18F-NaF uptake correlated closely with the change in calcium score at 1 year [141] ^18^F-NaF uptake (maximum tissue-to-background ratio 1·90 [IQR 1.61–2.17]) associated with ruptured plaques and those with high-risk features [142] Aneurysms in the highest tertile of ^18^F-NaF uptake expanded 2.5× more rapidly than those in the lowest tertile and were 3× more likely to rupture [143]	Single Multicentre	Coronary stenting, aneurysm stenting
	MTR	Q	Multiple sclerosis	Disease progression	MTR significantly correlates with T2 lesion volume [144] Grey matter MTR histogram peak height and average lesion MTR percentage change after 12 months independent predictors of disability worsening at 8 years [145] Change in brain MTR specificity 76.9% and PPV 59.1% for Expanded Disability Status Scale score deterioration [146]	Multicentre Single centre Single centre	Timing of therapeutic intervention
Malignant disease	^*18*^ *FDG-SUV*	Q	Cancer Oesophageal cancer	Good or poor prognosis tumour in terms of PFS and OS	Wide variation between individuals and tumours [147] Oesophageal cancer HR 1.86 for OS, 2.52 for DFS [148]	Meta-analysis	Neoadjuvant or adjuvant therapy or treatment modality combinations
	^18^FLT-SUV	Q	Cancer	High proliferative activity present	Sizeable overlap in values with normal proliferating tissues [75]	Review of data from single centre studies	Neoadjuvant or adjuvant therapy or treatment modality combinations
	*ADC* MRF (ADC, T1 and T2)	Q Q Q	Cancer, correlates with tumour grade	Risk of recurrence or metastasis	Area under ROC, sensitivity and specificity of nADCmean for G3 intrahepatic cholangiocarcinoma versus G1+G2 were 0.71, 89.5% and 55.5% [149] “Unfavourable” ADC in cervix cancer predictive of disease-free survival (HR 1.55) [150] ADC and T2 together give AUC of 0.83 for separating high- or intermediate-grade from low-grade prostate cancer [151]	Single centre Meta-analysis Single centre	Need of biopsy or other invasive diagnosis Neoadjuvant or adjuvant therapy Decision for radical treatment or active surveillance
	DSC-MRI	SQ (rCBV)	Brain cancer	Grading glioma	AUC = 0.77 for discriminating glioma grades II and III [152]	Meta-analysis	Type and time of intervention/treatment
	APT	Q	Glioma	Proliferation	APT correlates with tumour grade and Ki67 index [153]	Single centre	Therapeutic strategies
	DCE-CT parameters Blood flow, permeability	Q	Rectal cancer Lung cancer		Blood flow 75% accuracy for detecting rectal tumours with lymph node metastases [154] CT permeability predicted survival independent of treatment in lung cancer [155]	Single centre Single centre	Surgical dissection, adjuvant radiotherapy Adjuvant therapy
	DCE-MRI parameters	Q	Cervix cancer Endometrial cancer Rectal cancer Breast cancer	Risk of recurrence or metastasis, survival	Tumour volume with increasing signal is a strong independent prognostic factor for DFS and OS in cervical cancer [156] Low tumour blood flow and low rate constant for contrast agent intravasation (k_ep_) associated with high-risk histological subtype in endometrial cancer [157] K^trans^, K_ep_ and V_e_ significantly higher in rectal cancers with distant metastasis [158] Ktrans, iAUCqualitative and ADC predict low-risk breast tumors (AUC of combined parameters 0.78)	Single centre Single centre Single centre Single centre	Neoadjuvant, adjuvant or multimodality treatment strategies
	Radiomic signature [159]	Q	Multiple tumour types [160, 161]	Tumour with good or poor prognosis	Data endpoints, feature selection techniques and classifiers were significant factors in affecting predictive accuracy in lung cancer [162] Radiomic signature (24 selected features) is significantly associated with LN status in colorectal cancer [163]	Single centre Single centre	Neoadjuvant or adjuvant treatment, immunotherapy Lymph node dissection, adjuvant treatment

Biomarkers used visually in the clinic are given in italics, and those that are used quantitatively are in bold

*Abbreviations*: *ADC* apparent diffusion coefficient, *APT* amide proton transfer, *AUC* area under curve, *BI-RADS* breast imaging reporting and data systems, *CBV* cerebral blood volume, *CoV* coefficient of variation, *CR* complete response, *CT* computerised tomography, *DCE* dynamic contrast enhanced, *DFS* disease-free survival, *DOTATOC* DOTA octreotitide, *DOTATATE* DOTA octreotate, *DSC* dynamic susceptibility contrast, *ECG* electro cardiogram, *FDG* fluorodeoxyglucose, *FLT* fluoro thymidine, *HR* hazard ratio, *HU* Hounsfield unit, *ICC* intraclass correlation, *IQR* interquartile range, *LVEF* left ventricular ejection fraction, *MRF* magnetic resonance fingerprinting, *MRI* magnetic resonance imaging, *MTR* magnetisation transfer ratio, *NCCN* National Comprehensive Cancer Network, *OS* overall survival, *pCT* perfusion computerised tomography, *PERCIST* positron emission tomography response criteria in solid tumours, *PD* progressive disease, *PFS* progression-free survival, *PPV* positive predictive value, *PI-RADS* prostate imaging reporting and data systems, *PR* partial response, *PSMA* prostate-specific membrane antigen, *RECIL* response evaluation in lymphoma, *RECIST* response evaluation criteria in solid tumours, *ROC* receiver operating characteristic, *SD* stable disease, *SUV* standardised uptake value, *SWE* shear wave elastography, *US* ultrasound

For assessing treatment response (Table 3), the key element in biomarker selection relates to the type of treatment and expected pathological response. For non-targeted therapies, tissue necrosis to cytotoxic agents is expected, so biomarkers that read-out on increased free water (CT Hounsfield units) or reduced cell density (ADC) are most useful. With specific targeted agents (e.g. antiangiogenics), specific biomarker read-outs (perfusion metrics by US, CT or MRI) are more appropriate [185]. Both non-targeted and targeted agents shut down tumour metabolism, so that in glycolytic tumours, FDG metrics are exquisitely sensitive [186]. Distortion and changes following surgery, or changes in the adjacent normal tissue following radiotherapy [122], reduce quantitative differences between irradiated non-malignant and residual malignant tissue, so must be taken into account [187]. In multicentre trials, it is also crucial to establish the repeatability of the quantitative biomarker across multiple sites and vendor platforms for response interpretation [4].

**Table 3:** Imaging biomarkers for disease response assessment (semi-quantitative and quantitative) with examples of current evidence for their use that would support decision-making

	Biomarker	SemiQ/Q	Disease	Question answered	Utility of biomarker	Data from	Potential decision for
Non-malignant disease	Volumetric high resolution CT density (quantitative interstitial lung disease, QILD)	Q	Scleroderma	Response to cyclophosphamide	24-month changes in QILD scores in the whole lung correlated significantly 24-month changes in forced vital capacity (*ρ* = − 0.37), diffusing capacity (*ρ* = − 0.22) and breathlessness (*ρ* = − 0.26) [164]	Single centre	Continue, change or stop treatment
	**Left Ventricular ejection fraction** LVEF	Q	Pulmonary hypertension Myocardial ischaemia/infarction	Right and left cardiac sufficiency Improvement in cardiac function	Increases in 6-min walk distance were significant correlated with change in right ventricular ejection fraction and left ventricular end-diastolic volume [165] Monitoring cardiac function [166]	Multicentre Multicentre	Continue, change or stop treatment
Malignant disease	**RECIST**/morphological volume	Q	Cancer	Response	Current guidelines for response assessment [167]	Multicentre	Continue, change or stop treatment
	**PERCIST/**metabolic volume [168]	Q	Cancer	Response	Current guidelines for response assessment	Multicentre	Continue, change or stop treatment
	Scoring systems for disease burden	SQ	Multiple sclerosis Rheumatoid arthritis	Reduction in disease burden	Effects on MRI lesions over 6–9 months predict the effects on relapses at 12–24 months) [169] International consensus on scoring system [170]	Meta-analysis Review	Continue, change or stop therapy
	DSC-MRI	SQ (rCBV)	Brain cancer	Differentiation of treatment effects and tumour progression	In 2 meta-analyses MRI had high pooled sensitivities and specificities: 87% (95% CI, 0.82–0.91) to 90% (95% CI, 0.85-0.94) sensitivity and 86% (95% CI, 0.77–0.91) to 88% (95% CI, 0.83-0.92) specificity [171, 172]	Meta-analysis	Decision to treat
	^**18**^**F FDG-SUV**_**max**_ [173]	Q	Multiple cancer types	Response to therapy	Rectal cancer-pooled sensitivity, 73%; pooled specificity, 77%; pooled AUC, 0.83 [174] Intratreatment low SUV_max_ (persistent low or decrease of ^18^F-FDG uptake) predictive of loco-regional control in head and neck cancer [175]	Meta-analysis Meta-analysis	Continue, change or stop therapy
	*Deauville or RECIL score on* ^*18*^ *F-FDG-PET*	SQ	Lymphoma	CR, PR, SD or PD [176]	Assessment of tumour burden in lymphoma clinical trials can use the sum of longest diameters of a maximum of three target lesions [177]	Multicentre	Continue, change or stop therapy
	Targeted agents HER2 PSMA	SQ	Breast cancer [178] Prostate cancer [179]	Reduction in tumour cells expressing these antigens	Tumour receptor specific Effects of treatment on receptor expression	Single centre studies, review	Continue, change or stop therapy
	*ADC* [117]	SQ Q	Rectal cancer Breast cancer	Response to neoadjuvant chemotherapy Response to neoadjuvant chemotherapy	Additional value in both the prediction and detection of (complete) response to therapy compared with conventional sequences alone [180] After 12 weeks of therapy, change in ADC predicts complete pathologic response to neoadjuvant chemotherapy (AUC = 0.61, *p* = 0.013) [181]	Review Multicentre	Continue, change or stop therapy, proceed to surgery
	CT perfusion/blood flow	Q	Oesophageal cancer	Response to chemoradiotherapy	Multivariate analysis identified blood flow as a significant independent predictor of response [182]	Single centre	Further treatment
	DCE-MR parameters	Q	Multiple cancer types	Response to therapy	Particular benefit in assessing therapy response to antiangiogenic agents [183]	Review	Change therapeutic strategy
	CT density HU	Q	Gastrointestinal stromal tumours	Response to chemotherapy	Decrease in tumour density of > 15% on CT had a sensitivity of 97% and a specificity of 100% in identifying PET responders versus 52% and 100% by RECIST [184]		Continue, change or stop therapy

Biomarkers used visually in the clinic are given in italics, and those that are used quantitatively are in bold

*Abbreviations*: *ADC* apparent diffusion coefficient, *APT* amide proton transfer, *AUC* area under curve, *BI-RADS* breast imaging reporting and data systems, *CBV* cerebral blood volume, *CoV* coefficient of variation, *CR* complete response, *CT* computerised tomography, *DCE* dynamic contrast enhanced, *DFS* disease-free survival, *DOTATOC* DOTA octreotitide, *DOTATATE* DOTA octreotate, *DSC* dynamic susceptibility contrast, *ECG* electro cardiogram, *FDG* fluorodeoxyglucose, *FLT* fluoro thymidine, *HR* hazard ratio, *HU* Hounsfield unit, *ICC* intraclass correlation, *IQR* interquartile range, *LVEF* left ventricular ejection fraction, *MRF* magnetic resonance fingerprinting, *MRI* magnetic resonance imaging, *MTR* magnetisation transfer ratio, *NCCN* National Comprehensive Cancer Network, *OS* overall survival, *pCT* perfusion computerised tomography, *PERCIST* positron emission tomography response criteria in solid tumours, *PD* progressive disease, *PFS* progression-free survival, *PPV* positive predictive value, *PI-RADS* prostate imaging reporting and data systems, *PR* partial response, *PSMA* prostate-specific membrane antigen, *RECIL* response evaluation in lymphoma, *RECIST* response evaluation criteria in solid tumours, *ROC* receiver operating characteristic, *SD* stable disease, *SUV* standardised uptake value, *SWE* shear wave elastography, *US* ultrasound

## Advancing new quantitative imaging biomarkers as decision-support tools to clinical practice

To become clinically useful, biomarkers must be rigorously evaluated for their technical performance, reproducibility, biological and clinical validity, and cost-effectiveness [6]. Table 4 gives current recommendations for use of quantitative biomarkers as decision support tools.

**Table 4:** Recommendations for the use of quantitative imaging biomarkers as decision-support tools

Recommendation	Current evidence	Action needed
Consider need for quantitation in relation to the decision being made	Semi-quantitative imaging biomarkers are successfully used in many clinical pathways.	• Classification systems retain a subjective element that could benefit from standardisation and refinement. • Development of automated and thresholding would enable more quantitative assessments
Use validated IB methodology for semi-quantitative and quantitative measures	Many single and multicentre trials validating quantitative imaging biomarkers with clinical outcome now exist.	• Harmonisation of methodology • Standardised reporting systems
Establish evidence on the use of quantitation by inclusion into clinical trials	Clinical trials are usually planned by non-imagers. Integration of imaging biomarkers into trials is dependent on what is available routinely to non-imagers in the clinic, rather than exploiting an imaging technique to its optimal potential.	• Inventory of imaging biomarkers accessible through a web-based portal would inform the inclusion and utilisation of imaging biomarkers within trials (The European Imaging Biomarkers Alliance initiative). • Certified biomarkers conforming to set standards (Quantitative Imaging Biomarkers Alliance initiative)
Validate against pathology or clinical outcomes to make imaging a “virtual biopsy”	Several major databanks hold imaging and clinical or pathology data • CaBIG (USA) • UK MRC Biobank (UK) • German National Cohort Study (Germany)	• Large data collection for validation of imaging and pathology • Curation in imaging biobanks
Select appropriate quality assured quantitative IB	Trials with embedded QA/QC procedures have indicated good reproducibility of quantitative imaging biomarkers (e.g. EU iMi QuIC:ConCePT project)	• Ensure curation and archiving of longitudinal imaging data with outcomes within trials
Open-source interchange kernel	Low comparability between image-derived biomarkers if hardware and software of different manufacturers are used.	• Harmonisation of image acquisition and post-processing over manufacturers

Technical validation establishes whether a biomarker can be derived reliably in different institutions (comparability) and on widely available platforms. Provision must be made if specialist hardware or software is required, or if a key tracer or contrast agent is not licensed for clinical use. Reproducibility, a mandatory requirement, is very rarely demonstrated in practice [188] because inclusion of a repeat baseline study is resource and time intensive for both patients and researchers. Multicentre technical validation using standardised protocols may occur after initial biological validation (evidence that known perturbations in biology alter the imaging biomarker signal in a way that supports the measurement characteristics assigned to the biomarker). Subsequent clinical validation, showing that the same relationships are observed in patients, may then occur in parallel to multicentre technical validation.

Once a biomarker is shown to have acceptable technical, biological and clinical validation, a decision must be made to qualify the biomarker for a specific purpose or use. Increasingly, the role of imaging in the context of other non-imaging biomarkers needs to be considered as part of a multiparametric healthcare assessment. For example, circulating biomarkers such as circulating tumour DNA are often more specific at detecting disease but do not localise or stage tumours. The integration of imaging biomarkers with tissue and liquid biomarkers is likely to replace many traditional and more simplistic approaches to decision-support systems that are used currently.

The cost-effectiveness of a biomarker is increasingly important in financially restricted healthcare systems where value-based care is increasingly considered [189]. However, the information may be derived from scans done as part of the patients’ clinical work-up. Nevertheless, additional imaging/image processing is expensive compared to liquid- and tissue-based biomarkers. Costs can be off-set against the cost saving from the unnecessary use of expensive but ineffective novel and targeted drugs. Health economic assessment is therefore an important part of translating a new biomarker into routine clinical practice. In an era of artificial intelligence, where radiologists are faced with an ever-increasing volume of digital data, it makes sense to increase our efforts at utilising validated, quantified imaging biomarkers as key elements in supporting management decisions for patients.

## Data Availability

Not applicable

## Abbreviations

ADC: Apparent diffusion coefficient
APT: Amide proton transfer
AUC: Area under curve
CBV: Cerebral blood volume
CEST: Chemical exchange saturation transfer
CoV: Coefficient of variation
CR: Complete response
CT: Computerised tomography
DCE: Dynamic contrast enhanced
DFS: Disease-free survival
DOTATOC: DOTA octreotitide
DOTATATE: DOTA-octreotate
DSC: Dynamic susceptibility contrast
DWI: Diffusion-weighted imaging
ECG: Electrocardiogram
ESR: European Society of Radiology
FDG: Fluorodeoxyglucose
FLT: Fluorothymidine
HR: Hazard ratio
HU: Hounsfield unit
ICC: Intraclass correlation
IPF: Interstitial pulmonary fibrosis
IQR: Interquartile range
LVEF: Left ventricular ejection fraction
MATV: Metabolic active tumour volume
MRF: Magnetic resonance fingerprinting
MRI: Magnetic resonance imaging
MTR: Magnetisation transfer ratio
MTT: Mean transit time
NCCN: National Comprehensive Cancer Network
OS: Overall survival
pCT: Perfusion computerised tomography
PERCIST: Positron emission tomography response criteria in solid tumours
PD: Progressive disease
PFS: Progression free survival
PPV: Positive predictive value
PI: Peak intensity
PR: Partial response
PSMA: Prostate specific membrane antigen
QA: Quality assurance
QC: Quality control
RADS: Reporting and data systems (BI, breast imaging; LI, liver imaging; PI, prostate imaging; TI, thyroid imaging; VI, vesicle imaging)
RECIL: Response evaluation in lymphoma
RECIST: Response evaluation criteria in solid tumours
ROC: Receiver operating characteristic
ROI: Region of interest
RSNA: Radiological Society of North America
SD: Stable disease
SUV: Standardised uptake value
SWE: Shear wave elastography
TTP: Time to peak
US: Ultrasound
VOI: Voxel of interest
